# Draft Genome Sequence of *Aneurinibacillus tyrosinisolvens* LL-002^T^, Which Possesses Some Pseudouridine Synthases

**DOI:** 10.1128/genomeA.00529-15

**Published:** 2015-05-28

**Authors:** Taishi Tsubouchi, Shinro Nishi, Tadashi Maruyama, Yuji Hatada

**Affiliations:** Japan Agency for Marine-Earth Science and Technology, Yokosuka-shi, Kanagawa, Japan

## Abstract

We report the 5.7-Mb draft genome sequence of *Aneurinibacillus tyrosinisolvens* strain LL-002^T^, isolated from organic- and methane-rich sea sediments. The draft genome sequence of strain LL-002^T^ consists of 5,693,818 bp in 136 contigs, with a G+C content of 44.5%, 5,946 potential coding sequences (CDS), 2 rRNAs, and 39 tRNAs.

## GENOME ANNOUNCEMENT

Microbial RNA undergoes posttranscriptional modification by several enzymes during maturation. The most common modification to rRNA and tRNA is the conversion of specific uridine residues to pseudouridine (ψ) by pseudouridine synthases (1). Pseudouridine, which is the C-5–glycoside isomer of uridine, was identified as the “fifth nucleotide” in 1957 (2). Since then, more than 100 chemical variants were found in several RNA species, about 80% of which were variants in tRNA (http://mods.rna.albany.edu/). On the other hand, ψ synthases are the enzymes that catalyze the site-specific isomerization of uridine residues that are already part of an RNA molecule (3). In this reaction, no energy input is required and no cofactors are involved (4). The uracil base in uridine is linked through its N-1 position to the C-1′ position of the ribose, so the base in uridine possesses one hydrogen bond acceptor and one donor. Isomerization catalyzed by ψ synthase occurs when the uracil base is rotated 180° through an N-3–C-6 diagonal axis. In ψ, the C-5 position of uracil is linked to the C-1′ position of the sugar, resulting in an increase in hydrogen bonding capacity compared with that in uridine (5). *A. tyrosinisolvens* LL-002^T^ (6) possesses seven ψ synthases according to the genome analysis.

The draft genome sequencing of *A. tyrosinisolvens* LL-002^T^ was performed on an Ion Torrent PGM sequencer (Life Technologies) equipped with a 318 chip. Data from the genomic DNA library contained 1,960,572 reads and 532,539,090 nucleotide bases, with an average read length of 271.62 bp using 400-base chemistry. Assembly using Newbler Ver.2.6 (Roche Diagnostics, USA), which is a software package for *de novo* DNA sequence assembly, generated 136 contigs with a maximum and minimum contig size of 414,016 bp and 530 bp, respectively.

The draft genome comprising 5,693,818 nucleotides was annotated with the help of the GeneMark S (7) and the Rapid Annotations using Subsystems Technology (RAST) (8) servers. These annotation results indicate that strain LL-002^T^ possesses nine genes coding ψ synthases: MBEAT2_0006 and MBEAT2_0009 code TruA and TruB, which modify the uridyl residue (nucleotide positions 38, 39, 40, and 55 in *Escherichia coli*) of the tRNA molecules, and these two genes show 64.6% and 67.6% identity with those of *A. aneurinilyticus*, respectively. As for rRNA modification, three genes coding RsuA (MBEAT2_0001, -0004, and -0008) and four genes coding RluA (MBEAT2_0002, -0003, -0005, and -0007) are found. RsuA modifies the uridyl residue (nucleotide position 516 in *E. coli*) of the 16S rRNA molecules and RluA modifies one (nucleotide position 746 in *E. coli*) of the 23S rRNA molecules and one (nucleotide position 32 in *E. coli*) of the tRNA molecules.

### Nucleotide sequence accession numbers.

The draft genome sequence of *A. tyrosinisolvens* LL-002^T^ reported in this paper has been deposited to the DDBJ/EMBL/GenBank under the accession number BBWZ00000000 (the accession numbers of the contigs are BBWZ01000001 to BBWZ01000136) in BioProject PRJDB3821.
